# Central syntropic effects elicited by trigeminal proprioceptive equilibrium in Alzheimer’s disease: a case report

**DOI:** 10.1186/1752-1947-6-161

**Published:** 2012-06-26

**Authors:** Vincenzo De Cicco

**Affiliations:** 1 Department of Oral Science, University ‘G.D’Annunzio’, via dei Vestini, 31, Chieti, 66100, Italy

## Abstract

**Introduction:**

The presented patient, affected by Alzheimer’s disease, underwent neuropsychological evaluation and functional magnetic resonance imaging investigation under occlusal proprioceptive un-balance and re-balance conditions. Saccadic and pupillometric video-oculographic examinations were performed in order to detect connected trigeminal proprioceptive motor patterns able to interfere with reticular formation cerebellum functions linked to visual and procedural processes prematurely altered in Alzheimer’s disease.

**Case presentation:**

A 66-year-old Caucasian man, affected by Alzheimer’s disease and with a neuropsychological evaluation issued by the Alzheimer’s Evaluation Unit, underwent an electromyographic investigation of the masseter muscles in order to assess their functional balance. The patient showed a bilateral lack of all inferior molars. The extreme myoelectric asymmetry in dental occlusion suggested the rebalancing of masseter muscular functions through concurrent transcutaneous stimulation of the trigeminal nerve supramandibular and submandibular motor branches. The above-mentioned method allows detection of symmetric craniomandibular muscular relation that can be kept constant through the use of a cusp bite modeled on the inferior dental arch, called orthotic-syntropic bite. A few days later, the patient underwent a new neuropsychological investigation, together with a functional magnetic resonance imaging study, and saccadic, pupillometric video-oculographic examinations in occlusal un-balance and re-balance conditions.

**Conclusions:**

Comparative data analysis has shown that a re-balanced occlusal condition can improve a patient’s cognitive-attentive functions. Moreover, the saccadic and pupillometric video-oculographic investigations have proven useful both in analyzing reticulo-cerebellar subcortical systems, prematurely altered in Alzheimer’s disease, and in implementing neurological evaluations.

## Introduction

An increasing amount of evidence has pointed out the effects generated in aged rats by the loss of molar teeth, with reduction of spatial memory, acetylcholine release from the parietal cortex [1] and alteration of the septohippocampal cholinergic system [2], while Yamazaki *et al*. [3] have verified that the number of extracted teeth was directly proportional to the loss of spatial memory and to the reduction of trkB messenger ribonucleic acid (mRNA) levels. Furthermore, epidemiological surveys and cross-sectional studies in subjects ranging from 50 to 80 years old have demonstrated that reduced chewing ability or dysfunctional teeth induced senile processes or degeneration of hippocampal neurons, with a decrease of cognitive function and learning effect [4,5]. On the basis of these findings, studies have been carried out to verify whether the rebalance of myoelectric asymmetries in dental occlusion could modify cognitive-attentive parameters, even in a subject affected by Alzheimer’s disease (AD). In line with the above-mentioned findings, a case report random selection has envisaged only the bilateral lack of all inferior molars and a neuropsychological evaluation of average seriousness. Moreover, computerized saccadic and pupillometric video-oculographic evaluations were performed for their cognitive-attentive and mnemonic nature [6]. These examinations have also allowed for the analysis of the response of reticular formation and cerebellum nuclei connected with visio-motor and procedural processes, prematurely altered in AD [7,8].

## Case presentation

**Figure 1:**
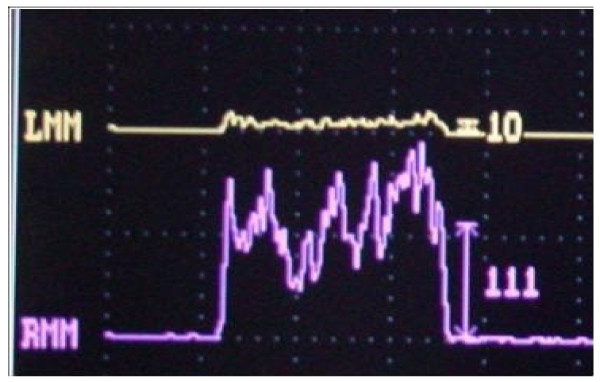
Electromyography (EMG) values of masseters in habitual occlusion: left myoelectric activity 10mV, right myoelectric activity 111mV.

**Figure 2:**
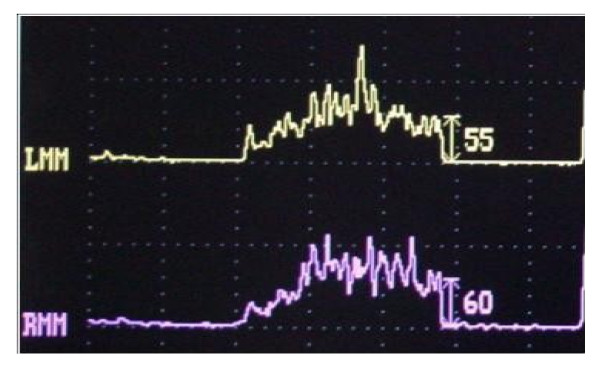
Electromyography (EMG) values of masseters in habitual occlusion: left myoelectric activity 55mV, right myoelectric activity 60mV.

The patient was a 66-year-old Caucasian man, who presented for a neuropsychological evaluation performed at the Centre of Alzheimer’s Evaluation Unit (AEU), with a Mini-Mental State Examination (MMSE) value of 18/30. The neuropsychological final report, as authentically quoted and faithfully pursuant to the description of the AEU doctor, related the following: ‘The present condition offers a picture of poor collaboration on the part of the patient who shows attention loss while performing the test and needs to be reminded about the given indications. Remarkable language worsening with reduced capacity of expression and comprehension that limit his autonomy. Worsening of executive functions with difficulty in planning and performing even simple activities, difficulty in solving problems. This picture defines a significant loss in instrumental activities for which assistance is needed. Treatment with anticholinesterasics and antioxidants must be continued’ (Table 1). For the evaluation of occlusal muscle activity, bilateral electromyography (EMG) of the masseter muscle was recorded using surface Ag/AgCl electrodes. In accordance with dental diagnostic protocols [9,10], a preliminary evaluation of the patient’s myoelectric activity in habitual dental occlusion was performed via electromyography of the muscles in order to assess their functional balance, as the patient showed a bilateral lack of all molars, second premolars and the right medial incisor. Registered values showed a remarkable functional asymmetry of the masseter muscles: 10 mV for the left masseter and 111 mV for the right masseter, respectively (Figure 1). According to the expressed electromyographic values, muscular activity was symmetrized by applying a 15-min transcutaneous stimulation of the trigeminal motor branches at low frequency for the masseter muscles and at medium frequency for the submandibular antagonist muscles. This method allowed detection of the functional trajectory of the occlusal elevator muscles and the recording of a symmetric craniomandibular relation by positioning a self-hardening material between the dental arches. The same material has been used successively to make a cusp bite modeled on the inferior dental arch, named the orthotic-syntropic bite for its particular use of electrostimulation. When the orthotic was applied, electromyographic control was repeated to verify the occlusal myoelectric balance. Substantially equal values were shown: 55 mV for the left masseter muscle and 60 mV for the right muscle (Figure 2).

**Table 1:** Neuropsychological values

**Category**	**Subcategory**	**Occlusal habitual**	**Orthotic-syntropic bite**
Orientation	Spatial	3/5	5/5
	Temporal	2/5	4/5
Memory	Immediate Rey test	11/75	15/75
	Deferred Rey test	3/15 7/15	
Reasoning	Raven matrices	20/36	22/36
Language	Token test	18/36	21/36
	Verbal flux per category	8	9
	Verbal flux per letter	14	13
Attention	Immediate visual memory	14/22	18/22
Apraxias	Ideational	17/20	18/20
	Constructional	12/20	16/20
Instrumental Activities of Daily Living (IADL)		4/8	6/8
Activities of Daily Living (ADL)		4/6	6/6
Mini-Mental State Examination (MMSE)		18/30	23/30

**Figure 3:**
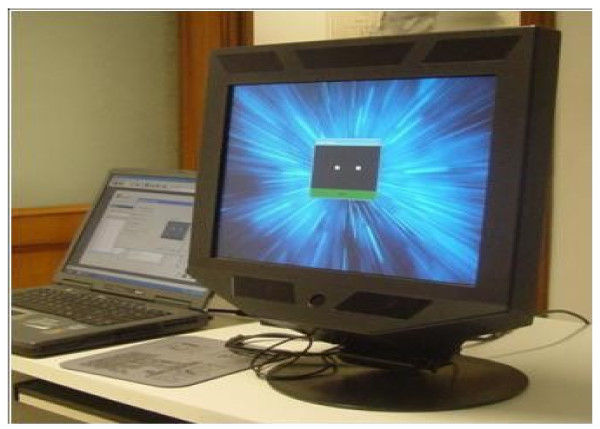
Video-oculographic system for saccadic test: the two white points that appear on the screen represent the fovea centralis retinae.

**Figure 4:**
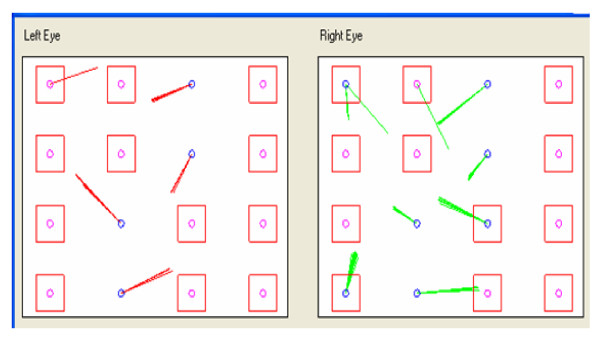
Video-oculographic saccadic test in habitual occlusion: 12 squares.

**Figure 5:**
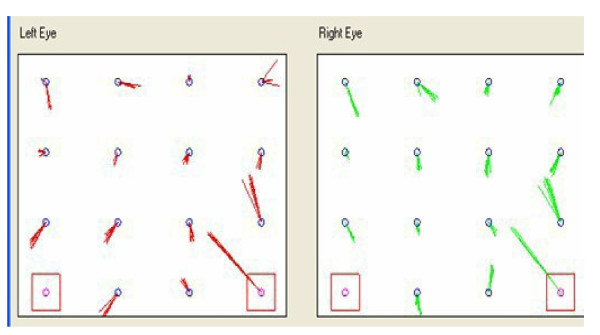
Video-oculographic saccadic test after orthotic-syntropic application: two squares.

**Figure 6:**
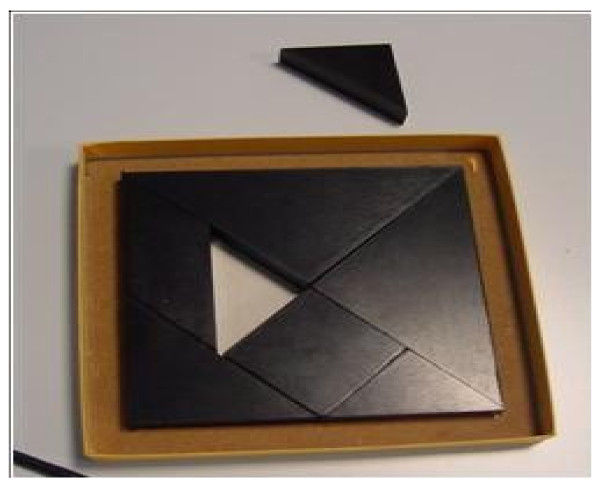
TanGram box with geometric forms for the cognitive task: the triangular form has been removed to show its specific place for repositioning.

**Figure 7:**
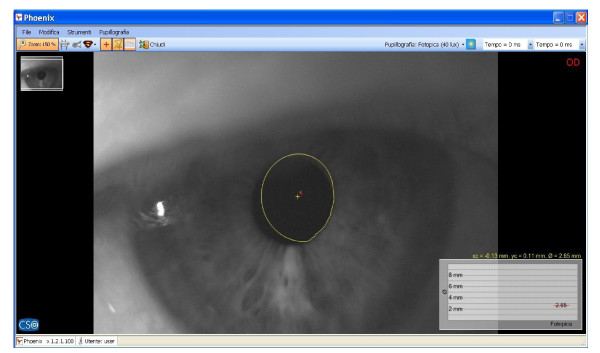
Basal pupillometry in habitual occlusion; pupil diameter: 2.65mm.

**Figure 8:**
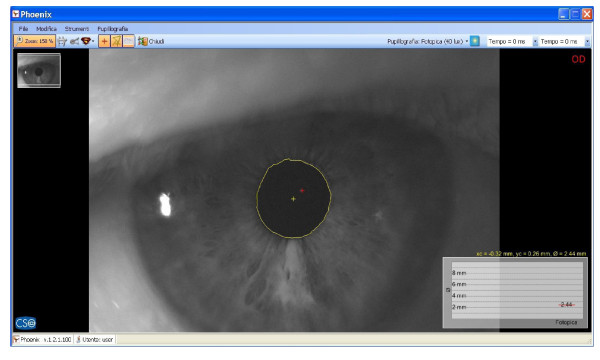
Pupillometry in TanGram test in habitual occlusion; pupil diameter: 2.44mm.

**Figure 9:**
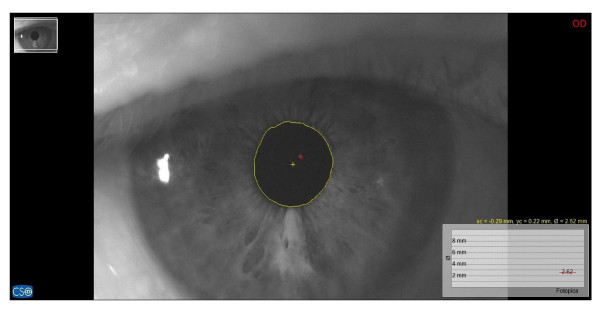
Basal pupillometry with orthotic-syntropic application; pupil diameter: 2.62mm.

**Figure 10:**
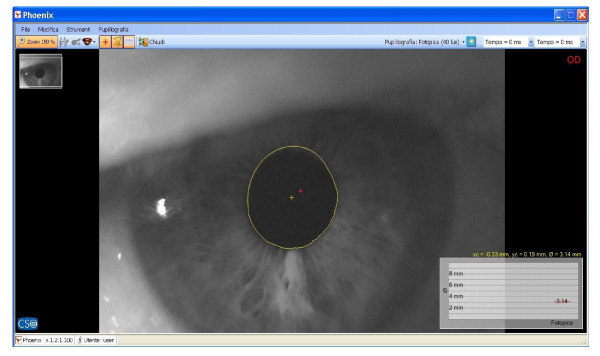
Pupillometry in TanGram test with orthotic-syntropic application; pupil diameter: 3.14mm.

Immediately after, saccadic, pupillometric and functional MRI (fMRI) examinations were performed, in habitual occlusion first and with the orthotic soon after; then, after a few days the patient was submitted again for neuropsychological evaluation. The saccadic video-oculography system used (Figure 3) is made up of a computerized eye-tracking system with eye tracking acquisition times of under 100 milliseconds. It is able to measure on a space-time diagram the distance between the point at which the patient is being observed and the point at which the target is situated in that exact moment. The system can evaluate the target/eye coherence for 16 points, showing hypometric and hypermetric mistakes with drifts of different extension and the total visual attentive incoherence of the patient with rectangles. The saccadic test calculates variable target routes that do not allow mnemonic effects. Indeed, in the occlusal un-balance condition, the video-oculographic program recorded complete target/eye inconsistency for 12 of the 16 detection points (Figure 4), while in the re-balance condition there were only two reported mistakes (Figure 5). Pupil diameter evaluations were measured with a corneal topographer made up of a survey section with a Placido disk (24 loops), charge-coupled device (CCD)1/3 camera sensor with 56mm working distance, and constant light and a chin support. The cognitive task given was a perceptive motor practognosic test, named TanGram, made up of a puzzle of triangular, square and parallelogram-shaped geometric forms (Figure 6). The patient, who had previously had how to perform the test explained to him, had to reposition in its specific place, without visual support, an element of the puzzle taken out and put in his right hand by the operator. The adopted protocol provided the pupillary diameter measurements (first basal and then during the cognitive task) just two seconds after the beginning of site exploration in the box. Pupillometric recordings were more interesting because when the patient performed the TanGram test, his basal pupillary diameter (2.65 mm) (Figure 7), which is physiologically inclined to extension, reduced by −0.21 mm (2.44 mm) (Figure 8), while in the occlusal re-balance condition a pupillometric increase of +0.58 mm (3.14 mm) (Figures 9 and 10) was registered, in line with what has been previously reported in the literature (Table 2). The results of fMRI performed in un-balance and re-balance conditions have been reported jointly and they precisely refer to: ‘type of performed exam: direct cerebral RM. Poorly collaborative patient. No significant areas of diffusion restriction. Some foci with gliotic signs related to old vascular problems can be detected in periventricular and subcortical areas. Insignificant signal alterations in the subtentorial area. Increase of ventricular cavities width, especially of the left occipital horn and of the subarachnoid spaces with an atrophic basis. It was not possible to perform a functional study with the activation of motor areas through right hand finger tapping due to the patient’s inability to perform the task correctly. After trigeminal stimulation and dental bite application, the functional study was made possible by an improved collaboration on the part of the patient, even if a correct activation of the pertinent motor area was not possible because the given orders were not performed at the same time as the acquisitions’.

**Table 2:** Computerized recordings of pupil diameters

	**Occlusal habitual**	**Orthotic-syntropic bite**
Basal, mm	2.65	2.62
TanGram test, mm	2.44	3.14

Finally, a neuropsychological report, undertaken after orthotic-syntropic bite application, related the following: ‘The neuropsychological evaluation evidences a higher collaboration of the patient, he is well oriented in space and shows light temporal disorientation. Minor difficulty in the comprehension of simple orders while the impediment to perform more complex tasks remains. The patient has improved short and medium term verbal memory span and his autonomy in performing instrumental activities of daily living, needing assistance only for more complex activities. Ideational slowdown remains. Improvement in executive functions capacities is evidenced, especially in planning, organization and problem solving abilities’, while the MMSE values in re-balance occlusion showed significant improvement (23/30), five points higher than the previous study (18/30) in the habitual condition (Table 1).

## Discussion

The results obtained in occlusal un-balance and re-balance conditions suggest that occlusal proprioceptive asymmetries can elicit central anisotropic effects characterized by minimum order configurations and minimum cortical-subcortical functional differentiations. Even if references to the literature are only indirect at the moment, in this case report the performed tests permit us to deduce that occlusal proprioceptive re-balance in the short/medium term can alter some central functional parameters. Overall, data analysis suggests that consistent effects are seen in the visual-spatial context, in the planning and execution of organization skills, while other functions, such as ideation, reasoning and execution of complex tasks, language and grammar complexity do not display significant results. Improved collaboration on the part of the patient during neuropsychological evaluation and in fMRI execution is a very significant element to take into account. With respect to this, the pupillometric exam can contribute to our understanding of the behavioral change on the part of the patient, because pupillary diameter variation represents an unequivocal evaluative element of the cognitive control state during an evoked task and it is strictly related to locus coeruleus (LC) tonic/phasic activity [11]. In fact, Cohen *et al*. have demonstrated that during task execution the anterior cingulate, orbitofrontal and prefrontal cortex stimulates LC phasic mode with norepinephrine release. This determines concomitant and immediate pupillary diameter increase, proportional to the released noradrenergic quantity [12]. A pupillometric reduction of −0.21 mm registered in occlusal un-balance during the test (Figures 7 and 8) may be interpreted as the result of cortical strain [13], while a pupillometric increase of +0.58 mm registered in occlusal re-balance, with a basal pupillometric value of 2.62 mm (Figures 9 and 10), is an index of unquestionable higher coerulean phasic expressivity. This is surprising because the LC is prematurely and deeply interested by AD degenerative processes [14], and it is also interesting with regard to basal reduction. Trigeminal neurophysiological mechanisms at the core of pupillometric clinical evidence cannot be exhaustively delineated at present, but some relationships among the trigeminal complex, coerulean system and reticular formation can be hypothesized. The literature mainly relates the projections and effects of the LC-norepinephrine system on trigeminal sensorimotor nuclei. Previous studies performed through anterograde and retrograde transport analysis have indicated that many of the regions that received dense inputs from the projected LC neurons, in turn, back upon these coerulei neurons [15], which are uniformly sensitive to a variety of non-noxious stimuli, including tactile, visual, auditory and taste with specific degree of activation stimulus, [16,17]. The trigeminal system is strictly connected with the LC and several works have proved that clusters of mesencephalic neuronal branches reach LC-pars compacta, which exhibit a mixture of cellular elements with trigeminal mesencephalic neurons, [15,18]. Couto et al. demonstrated with retrograde tract tracing using fast blue injections in spinal and principal sensory trigeminal nuclei, the presence of labeled trigeminal mesencephalic and cerulean neurons, [19]. Moreover, Panneton *et al*. proved trigemino-autonomic connections, using herpes simplex virus 1 (HSV-1) (strain 129), with an anterograde transneuronal transport method that LC and paragigantocellularis nuclei were also labeled [20]. Seemingly, the LC can be activated by increasing the discharge frequency of trigeminal mesencephalic neurons activated both by masseter spindle receptors due to interocclusal excessive space [21], and by the periodontal for increased occlusal charge, with glutamate release for the activation of presynaptic γ-aminobutyric acid (GABA_A_) receptors, on the coerulean and peri-coerulean zone [22]. These conditions, characterized by neuromotor facilitation of the mastication preferential side, are inevitably associated with contralateral functional hypoactivity of the trigeminal nerve motor and mesencephalic nuclei. Occlusal motor-proprioceptive activity probably produces a concomitant and homolateral asymmetry of LC/noradrenaline (LC-NE) system phasic modes. Specifically, we may believe that occlusal balance symmetrization can determine, in the trigeminal/LC-NE mesencephalic nucleus pathway, a coerulean activation on the hypoactive occlusal side and a concomitant contralateral reduction which, moreover, could also determine a lower galanin release, normally hyperexpressed in AD, from noradrenergic terminations [23,24]. In fact, Hoogendijk *et al*. have demonstrated through the determination of NE and of its 3-methoxy-4-hydroxyphenylglycol (MHPG) metabolite in different brain areas that a significant reverse relationship between the number of coerulean neurons and MHPG/NE ratio both in frontal cortex and in LC can be found in subjects affected by AD, while a significant rise of the MHPG/NE ratio indicates a consistently increased metabolism [25]. In addition to this hypothesis, the coerulean area can also be indirectly activated by the trigeminal motor nucleus. This nucleus does not have a definite nuclear delimitation but it is mixed with lateral reticular formation (LRF) parvocellular neurons [26], and it is part of the ascending reticular activating system [27]. Presumably, neuromotor hyperactivity of the mastication preferential side elicits a concomitant asymmetric brainstem stimulation reward [28], including diffused projection catecholaminergic systems of intermediate reticular formation nuclei (IRFn). Previous research has recorded short latency hypsilateral orthodromic responses in LRF and IRFn after electrostimulation of the masseteric nerve and after passive mandible dislocations, [29]. Therefore, a hypothesis can be made that the pupillary diameter increase (3.14 mm) (Figure 10) registered in occlusal re-balance during the evoked task may be the result of a more effective and synchronous phasic expressivity of cerulean neurons, associated with a more suitable reduction (2.62 mm) (Figure 9) of the basal diameter. This last result further validates the functional relationships between coerulean and trigeminal systems because, on the one hand, Rajkowski *et al*. have demonstrated that basal pupillary diameter is strictly connected to LC tonic discharge frequency. On the other hand, Yabushita *et al*. have shown that the occlusal vertical dimension increase, which we obtained by orthotic syntropic application, reduces neuromuscular spindle discharge frequency of the masseter muscles [21]. The cited research also suggests that an increased inter-occlusal free space inevitably implies extreme masseter muscle contraction during occlusion in swallowing, determining an increase of spindle and periodontal discharge frequency that hyperactivates the mesencephalic nucleus with glutamate, having depolarizing functions on cerulean neurons. The final result is an increased LC tonic activity that can be detected in the pupillary basal diameter size. The results obtained from saccadic and pupillometric tests confirm the above. Reports in the literature state that saccadic final control is developed by perfect coordination and synchrony of both prepositus hypoglossi and paramedian pontine reticular nuclei [30,31], and the ipsicerebellar fastigial contralateral to saccadic movement nuclei [8]. Fastigial nuclei can certainly be a target of occlusal asymmetric motor activity, especially of the hypofunctional factor, since the brainstem burst generator cannot produce accurate saccades without oculomotor cerebellum contribution [32]. In fact, these nuclei operate a codification of saccadic command space-time transformation through absolute functional synchrony of the contralateral (initial facilitation of the ‘burst’ scale) and ipsilateral (late ‘burst’ discharge inhibition) fastigial nuclei to saccadic movement and their diminished cooperation can determine saccadic hypo/ipermetry [33]. At present, it is not possible to confirm if the targets of occlusal asymmetry are mainly reticular nuclei, cerebellum fastigial nuclei, or both, but the radical improvement registered in the saccadic test after occlusal re-balance can be interpreted, in apparent contrast with the high stability of the neuronal systems controlling and programming it, as an index of reticulo-cerebellar functional synchrony, equal to what has been hypothesized for coerulean neuron activation modes.

## Conclusions

The results of this case report suggest that occlusal un-balance may represent an interferential pattern in some AD central functions. Pupillary dynamic and saccadic motor control examinations can be an efficient investigation tool for implementing neurologic evaluations, and identifying patterns interfering with tonic/phasic LC modes. Within the limits of this case report, further investigations are necessary in order to detect the modalities by which the muscular and periodontal proprioception may modulate the cognitive attentive activity.

## Consent

Written informed consent was obtained from the patient’s next of kin for publication of this manuscript and any accompanying images. A copy of the written consent is available for review by the Editor-in-Chief of this Journal.

## Competing interests

The author declares that he has no competing interests.
